# Dataset on Green IT adoption in Indonesian higher education institutions: A survey-based study

**DOI:** 10.1016/j.dib.2026.112891

**Published:** 2026-05-26

**Authors:** Uky Yudatama, Pristi Sukmasetya, Agus Setiawan, Endah Ratna Arumi

**Affiliations:** Department of Informatics, Faculty of Engineering, Universitas Muhammadiyah Magelang, Magelang, Indonesia

**Keywords:** Green IT implementation, Higher education sustainability, Environmental IT strategies, Green IT, Green technology

## Abstract

This dataset was collected using a detailed and rigorous methodology to examine Green IT practices in the Indonesian higher education sector. A structured online questionnaire was distributed to various institutions through a secure digital platform, targeting academics with expertise in IT and sustainability. A random sampling method was employed to ensure a diverse and representative sample of respondents. This approach facilitated high participation rates and enabled the collection of standardized data, ready for immediate analysis, thereby enhancing the quality and reliability of the findings. The dataset comprises quantified responses captured through Likert scales, focusing on multiple dimensions of Green IT, including compliance, awareness, challenges, and benefits. This structured approach ensured precise measurement of attitudes and perceptions of Green IT practices, minimizing variability that might arise from open-ended questions. The comprehensive nature of the data, gathered from individuals with diverse demographic and professional backgrounds, provides robust insights into the factors influencing Green IT implementation in the educational sector. The dataset’s structured format and depth of information offer substantial potential for reuse in future research. It can serve as a benchmark for comparative studies, support longitudinal research to monitor changes over time or aid in developing targeted interventions to enhance Green IT practices in higher education institutions. Overall, this dataset contributes significantly to the academic discourse on Green IT and provides actionable insights for policymakers and institutional leaders striving to promote more sustainable IT practices within the educational sector.

Specifications Table

**Table:** 

Subject	Information System
Specific subject area	Green Information Technology, Factors, Influence Factors, and Connectivity
Type of data	Table
Data collection	Data were collected using an online questionnaire distributed among academics familiar with Green IT practices in higher education. The questionnaire was developed based on a comprehensive literature review of Green IT implementation in universities, ensuring relevance and thorough coverage of the topic. The questions were designed to be specific and clear, incorporating verbal labels for response options to minimize ambiguity and enhance response quality. Each item focused on a single idea to avoid confusion and improve the accuracy of responses. Random sampling methods were employed to increase the representativeness of the sample within the academic community. This methodological approach aimed to capture a diverse range of insights into the adoption and challenges of Green IT across higher education institutions in Indonesia.
Data source location	Big cities: Jakarta, Banten, Bandung, Yogyakarta, Semarang, Surabaya, Medan, Aceh. Country: Indonesia
Data accessibility	The data is available via Mendeley. Yudatama, Uky (2025), “Dataset Green IT Adoption in Sector Indonesian Higher Education”, Mendeley Data, V2, doi: 10.17632/tdd78ttwkd.2 It can be accessed via this link: https://data.mendeley.com/datasets/tdd78ttwkd/2

## 1 Value of the Data

• **Identifying Key Success Factors:** This data set can pinpoint crucial factors that influence the success of Green IT implementation in Indonesian universities. By analyzing responses, researchers can identify which resources, policies, or practices are most strongly correlated with successful Green IT outcomes. This is valuable for universities aiming to optimize their strategies for sustainability in technology.
• **Interrelationships Between Factors:** By examining how different factors such as budget allocations, institutional commitment, and regulatory support interact, researchers can understand the dynamics that enhance or hinder Green IT initiatives. This can lead to more effective strategies that leverage positive interactions and mitigate negative ones.
• **Impact Analysis of Influential Factors:** The data allows for the analysis of which factors have the greatest impact on the success of Green IT implementation. Researchers can use statistical tools to quantify the influence of each factor, providing empirical evidence to guide future decisions in university policy and IT strategy development.
• **Policy Development Support:** Insights from the data can support the development of policies that foster an environment conducive to Green IT practices. Understanding which factors are most influential helps in prioritizing policy interventions that could lead to more successful implementations.
• **Resource Allocation Guidance:** For university administrators and decision-makers, understanding the key factors influencing Green IT success can inform more strategic allocation of resources. This might include investing in specific technologies, training programs, or sustainability initiatives that are shown to be most effective.
• **Novel Measurement Contribution:** This dataset introduces a newly developed and context-specific measurement instrument for assessing Green IT implementation in higher education. The instrument integrates multiple dimensions-such as policy, management commitment, awareness, infrastructure, and financial support-into a comprehensive framework. As such, it provides a valuable foundation for future research aiming to measure Green IT adoption, particularly in developing country contexts. Researchers may adapt, validate, or extend this instrument in different institutional or geographical settings.

## 2 Background

In Indonesia's higher education sector, the implementation of Green IT is challenged by financial constraints, weak institutional commitment, and inadequate regulatory support [1]. Many state-run universities face budgetary limitations that prevent updates to more efficient, environmentally friendly technologies [2]. This financial issue is prevalent across developing nations and slows the adoption of sustainable technological practices [3,4]. Additionally, strategic planning for sustainability and green technologies often takes a backseat, further inhibiting the widespread deployment of Green IT [5]. The lack of firm administrative commitment and clear regulatory guidelines exacerbates these challenges, making it difficult to enforce sustainability policies effectively [[5], [6], [7]]. Furthermore, there is a significant gap in awareness and understanding of the benefits of Green IT among students and faculty in Southeast Asia [[7], [8], [9]]. This is compounded by a shortage of IT professionals with expertise in green technology management, which is crucial for the successful implementation of Green IT initiatives in the region [2,8,9].

## 3 Data Description

The dataset comprises a comprehensive collection of responses from academics familiar with Green IT practices in higher education institutions across Indonesia. It is systematically organized into several sections, each designed to capture specific aspects of Green IT implementation and its influencing factors. The first section contains demographic information, including details on respondents' gender, age, highest educational attainment, and current institutional roles. This information provides a foundational understanding of the participant pool's diversity and serves as a context for interpreting the rest of the data. The second section delves into the professional backgrounds of the respondents, focusing on their experience in the IT field, with particular attention to Green IT. This section aims to establish a correlation between the respondents' level of experience and their perspectives on the effectiveness of Green IT practices, offering insights into how professional expertise influences implementation. The third section examines the characteristics of the institutions represented, differentiating between public and private entities while assessing their commitment to Green IT. This section facilitates an analysis of how institutional type and varying levels of commitment impact the adoption and success of Green IT initiatives. Responses related to Green IT policies and compliance form the fourth section. It includes data on the existence of formal green IT policies, the institutions' compliance with environmental regulations, and the alignment of Green IT initiatives with institutional missions. This part of the dataset is crucial for understanding the structural and policy-driven elements that support or hinder Green IT practices. The fifth section delves into management involvement, highlighting the role of leadership in fostering Green IT. It examines the commitment of top management to Green IT, their involvement in strategic decision-making, and the resources allocated to Green IT efforts. Leadership's role is scrutinized to understand its impact on the success of Green IT initiatives.

The sixth section assesses awareness and education regarding Green IT within the institutions. It gathers responses on the awareness of Green IT's importance and the presence of training programs aimed at enhancing this understanding. This data is vital for evaluating how well-prepared institutions are to undertake and sustain Green IT practices. Technology usage and infrastructure is the focus of the seventh section. It covers the application of energy-efficient technologies, the maintenance of Green IT infrastructure, and the degree to which these practices are integrated into the daily operations of the institutions. This section provides a practical view of the tangible actions taken by institutions to implement Green IT. Overall, this dataset is meticulously organized to facilitate a comprehensive analysis of Green IT in higher education. By examining these varied yet interconnected data points, researchers can gain a holistic understanding of the factors that drive or impede the implementation of sustainable IT practices in educational institutions. Each section of the dataset not only stands alone in offering insights into specific aspects but also contributes to a collective understanding of the broader Green IT landscape in Indonesia's higher education sector.

The demographic profile of respondents in Table 1 shows that most respondents are male, 54.5% (150 respondents), while females are 45.5% (125 respondents). Regarding age, most respondents are in the 25-34 age range, which is 43.6% (120 respondents). Followed by the 18-24 age group at 25.5% (70 respondents), the 35-44 age group at 21.8% (60 respondents), and those aged 45 years and above at 9.1% (25 respondents). Regarding education, the largest proportion of respondents have a bachelor’s degree (43.6%, 120 respondents), followed by those with Diploma qualifications (25.5%, 70 respondents). Respondents with postgraduate qualifications (S2/S3) reached 16.4% (45 respondents), while 14.5% (40 respondents) have completed high school or equivalent education. In short, respondents were mostly young adults, mainly male, with a strong educational background, especially at the bachelor’s level.

**Table 1:** Demographic information

Demographic		N	Percentage
**Gender**	Man	150	54.5
	Woman	125	45.5
			
**Age Group**	18 - 24 years old	70	25.5
	25 - 34 years old	120	43.6
	35 - 44 years old	60	21.8
	45 years and older	25	9.1
			
**Education Level**	High School/Equivalent	40	14.5
	Diploma	70	25.5
	Bachelor (S1)	120	43.6
	Pascasarjana (S2/S3)	45	16.4

Table 2 outlines the variables used providing detailed descriptions of each. The ID variable serves as a unique numeric identifier for each respondent. The remaining variables are measured on a 5-point ordinal scale, ranging from 1 (Strongly Disagree) to 5 (Strongly Agree). These variables include Policies and Regulations (PR), which evaluates perceptions of regulatory frameworks, and Commitment and Management Support (CM), which assesses the level of managerial support. Awareness and Education (AE) measures the respondents' awareness and educational initiatives related to Green IT, while Infrastructure and Technology (IT) reflects the adequacy of infrastructure supporting these initiatives. Additionally, Business and Operational Processes (BO) examines operational practices, and External and Social Pressure (ES) captures the influence of external and societal factors. Finance and Investment (FI) measures perceptions of financial support allocated to Green IT, and finally, Green IT Success (GS) evaluates the overall success of Green IT implementations. All variables, except the respondent ID, are ordinal and have no missing values, offering a structured framework for analyzing factors that contribute to the success of Green IT initiatives.

**Table 2:** Measurement constructs and item descriptions

Construct	Code	Description	Sample Item
Policies and Regulations	PR	Institutional policies supporting Green IT	“Our institution has formal policies supporting Green IT.”
Commitment and Management Support	CM	Top management commitment and support	“Top management actively supports Green IT initiatives.”
Awareness and Education	AE	Awareness and training on Green IT	“Staff and students are aware of Green IT importance.”
Infrastructure and Technology	IT	Availability of Green IT infrastructure	“Energy-efficient technologies are implemented.”
Business and Operational Processes	BO	Integration into daily operations	“Operational processes include Green IT practices.”
External and Social Pressure	ES	External influence and pressure	“External stakeholders encourage Green IT adoption.”
Finance and Investment	FI	Financial support for Green IT	“Sufficient budget is allocated for Green IT.”
Green IT Success	GS	Effectiveness of implementation	“Green IT initiatives have been successful.”

The measurement instrument used in this study was developed based on an extensive review of prior literature on Green Information Technology (Green IT), sustainability practices, and technology adoption in higher education. The questionnaire consists of multiple subscales representing key latent constructs: Policies and Regulations (PR), Commitment and Management Support (CM), Awareness and Education (AE), Infrastructure and Technology (IT), Business and Operational Processes (BO), External and Social Pressure (ES), Finance and Investment (FI), and Green IT Success (GS). Each construct is operationalized using multiple reflective indicators measured on a five-point Likert scale ranging from 1 (“Strongly Disagree”) to 5 (“Strongly Agree”).

The Policies and Regulations (PR) construct assesses the extent to which institutional policies support Green IT implementation. Sample item: “Our institution has formal policies supporting environmentally sustainable IT practices.” Commitment and Management Support (CM) captures top management involvement and strategic commitment. Sample item: “Top management actively supports Green IT initiatives.” Awareness and Education (AE) measures the level of awareness and training related to Green IT. Sample item: “Staff and students are aware of the importance of Green IT practices.” Infrastructure and Technology (IT) evaluates the availability and adequacy of environmentally friendly IT infrastructure. Sample item: “Energy-efficient technologies are implemented in our institution.” Business and Operational Processes (BO) reflects the integration of Green IT into daily operations. Sample item: “Operational processes incorporate environmentally sustainable IT practices.” External and Social Pressure (ES) captures external influences such as societal expectations or regulatory demands. Sample item: “External stakeholders encourage the adoption of Green IT.” Finance and Investment (FI) measures financial readiness and investment in Green IT. Sample item: “Sufficient budget is allocated for Green IT implementation.” Green IT Success (GS) evaluates the perceived effectiveness of Green IT implementation. Sample item: “Green IT initiatives in our institution have been successfully implemented.”

Composite scores for each construct were calculated by averaging the scores of their respective items, rather than summing them, to maintain consistency with the original Likert scale metric. This approach allows for easier interpretation and comparability across constructs. All indicators were treated as reflective measures, consistent with the theoretical assumption that observed items reflect the underlying latent constructs. All items in the questionnaire were positively worded; therefore, no reverse-coded items were included in the instrument. Consequently, no reverse scoring was required during data preprocessing. The dataset provided has already been cleaned and is ready for analysis, with all item scores aligned in the same direction, where higher values indicate stronger agreement and more favorable perceptions of Green IT practices.

The measurement model was evaluated using standard PLS-SEM criteria, including indicator loadings, Cronbach’s alpha, composite reliability (CR), and average variance extracted (AVE). Most constructs demonstrated acceptable reliability (α > 0.7) and convergent validity (AVE > 0.5), indicating that the measurement instrument is generally robust, although several indicators with lower loadings suggest opportunities for refinement. As this study employs a newly developed measurement instrument tailored to the Indonesian higher education context, the inclusion of multiple items per construct and the use of established validation procedures are intended to ensure both conceptual coverage and empirical reliability. Future studies are encouraged to further validate and refine this instrument across different contexts.

**Fig. 1:**
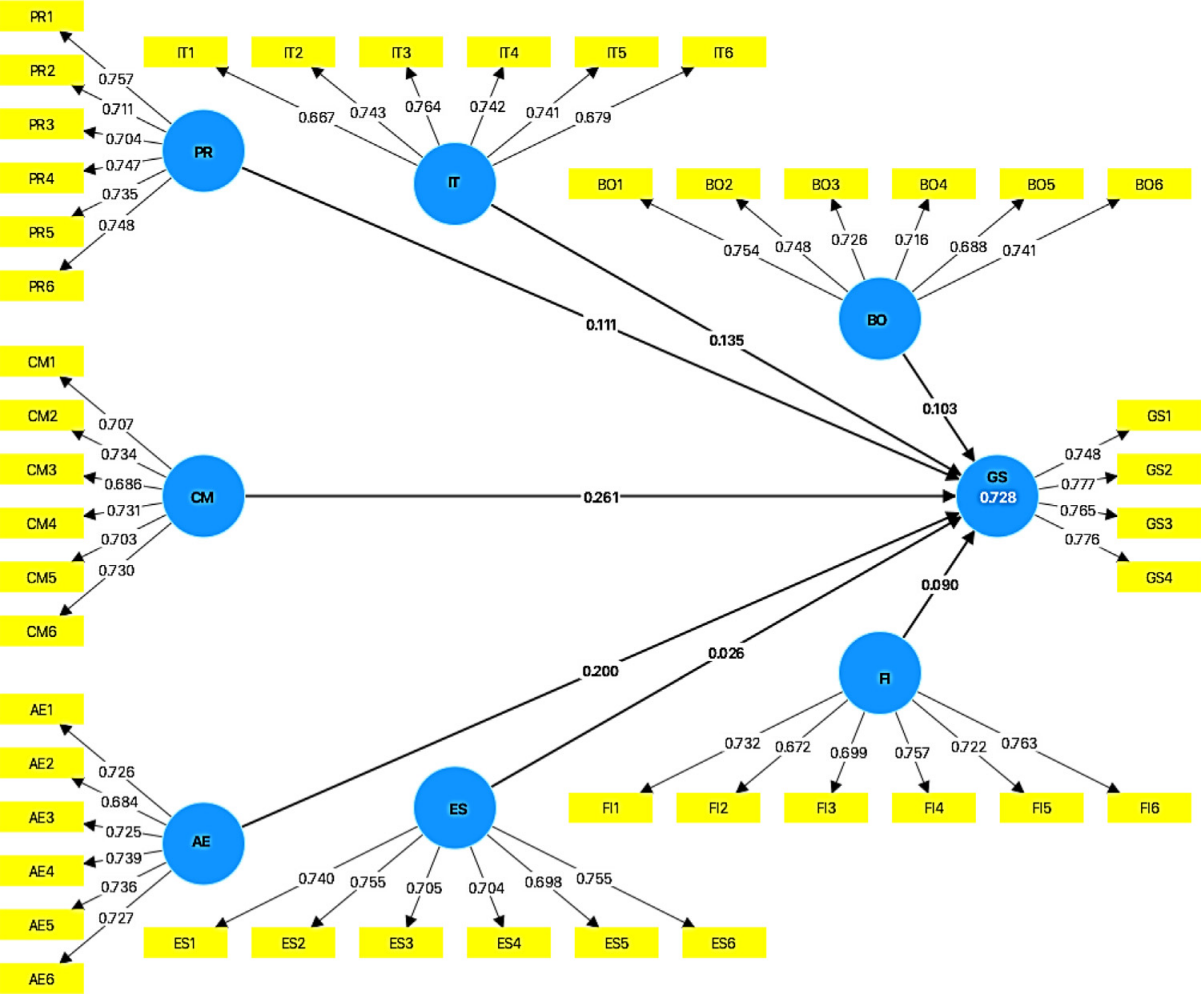
Structural model

Fig. 1 presents the structural model illustrating the relationships between latent variables, including Policies and Regulations (PR), Commitment and Management Support (CM), Awareness and Education (AE), Infrastructure and Technology (IT), Business and Operational Processes (BO), External and Social Pressure (ES), Finance and Investment (FI), and Green IT Success (GS).

The model includes path coefficients representing the relationships between constructs. The largest coefficient observed is between CM and GS (0.261), followed by BO (0.103) and FI (0.090) toward GS. Other coefficients include PR→IT (0.111), AE→ES (0.200), and ES → FI (0.026).

These values ​​are presented to describe the structure of the dataset and the relationships among variables, without implying causal conclusions.

Table 3 shows the convergent validity analysis of various constructs in the model based on factor loading values, Cronbach's alpha (α), Average Variance Extracted (AVE), and Composite Reliability (CR). In general, constructs such as Awareness and Education (AE), Business and Operational Processes (BO), Commitment and Management Support (CM), External and Social Pressure (ES), Finance and Investment (FI), Infrastructure and Technology (IT), Policies and Regulations (PR), and Green IT Success (GS) show good validity and reliability. Most constructs have Cronbach's alpha values ​​above 0.7, indicating a high level of reliability. In addition, the AVE values ​​for all constructs exceed 0.5, which means that the indicators used can explain the latent variables well. Composite Reliability (CR) for all constructs is also above 0.7, indicating good internal consistency. However, several indicators show factor loading below 0.7, such as AE2, CM3, ES5, FI2, FI3, IT1, and IT6. This indicates that these indicators are not strong enough to explain their respective constructs and may require revision or evaluation. The constructs with the best performance are Policies and Regulations (PR) and Business and Operational Processes (BO), which show very good validity and reliability values. Meanwhile, Finance and Investment (FI) and Infrastructure and Technology (IT) have several weak indicators and require more attention to improvement.

**Table 3:** Convergent validity based on factor loading, Cronbach’s alpha, AVE, and CR

Constructs	Items	Factor Loading	λ Cronbach’s alpha	α AVE	CR
**Awareness and Education (AE)**	AE1	0.726	0.817	0.523	0.868
	AE2	0.684			
	AE3	0.725			
	AE4	0.739			
	AE5	0.736			
	AE6	0.727			
**Business and Operational Processes (BO)**	BO1	0.754	0.824	0.531	0.872
	BO2	0.748			
	BO3	0.726			
	BO4	0.716			
	BO5	0.688			
	BO6	0.741			
**Commitment and Management Support (CM)**	CM1	0.707	0.809	0.512	0.863
	CM2	0.734			
	CM3	0.686			
	CM4	0.731			
	CM5	0.703			
	CM6	0.730			
**External and Social Pressure (ES)**	ES1	0.740	0.821	0.528	0.870
	ES2	0.755			
	ES3	0.705			
	ES4	0.704			
	ES5	0.698			
	ES6	0.755			
**Finance and Investment (FI)**	FI1	0.732	0.819	0.525	0.869
	FI2	0.672			
	FI3	0.699			
	FI4	0.757			
	FI5	0.722			
	FI6	0.763			
**Infrastructure and Technology (IT)**	IT1	0.667	0.817	0.523	0.868
	IT2	0.743			
	IT3	0.764			
	IT4	0.742			
	IT5	0.741			
	IT6	0.679			
**Policies and Regulations (PR)**	PR1	0.757	0.828	0.538	0.875
	PR2	0.711			
	PR3	0.704			
	PR4	0.747			
	PR5	0.735			
	PR6	0.748			
**Green IT Success (GS)**	GS1	0.748	0.766	0.588	0.851
	GS2	0.777			
	GS3	0.765			
	GS4	0.776			

Overall, this model is quite good at meeting the validity and reliability criteria, but there is still room to improve the quality of the model, especially in indicators with low factor loading. Revision of these indicators can strengthen the analysis results and reliability of the model.

The assessment of discriminant validity using the Fornell-Larcker criterion in Table 4 shows that, although the square root of the AVE for each construct is generally higher than its correlation with other constructs, some inter-construct correlations remain relatively high (e.g., between FI-IT and PR-BO). This indicates potential overlap between certain constructs.

**Table 4:** Discriminant Validity-Fommel Larcker criterion

	AE	BO	CM	ES	FI	GS	IT	PR
**AE**	0.723							
**BO**	0.828	0.729						
**CM**	0.777	0.816	0.715					
**ES**	0.810	0.808	0.815	0.727				
**FI**	0.835	0.831	0.807	0.823	0.725			
**GS**	0.786	0.781	0.796	0.760	0.780	0.767		
**IT**	0.813	0.818	0.807	0.815	0.835	0.781	0.723	
**PR**	0.821	0.836	0.830	0.828	0.833	0.786	0.822	0.734

Therefore, although the model meets the minimum threshold for discriminant validity, the results should be interpreted with caution, as distinctions between some constructs may not be entirely clear. This overlap suggests that certain dimensions of Green IT implementation in higher education are conceptually and empirically interrelated, rather than completely independent.

Consequently, the discriminant validity in this study can be considered acceptable but not optimal, and this limitation should be recognized when interpreting the structural relationships.

Table 5 presents the path coefficients along with their corresponding T-statistics and p-values. For example, the AE → GS relationship shows a coefficient of 0.200 (T = 2.675, p = 0.007), while CM → GS shows a coefficient of 0.261 (T = 3.964, p = 0.000).

**Table 5:** Total effects-mean, STDEV, T values, p values

	Original sample (O)	Sample mean (M)	Standard deviation (STDEV)	T statistics (|O/STDEV|)	P values
**AE** → **GS**	0.200	0.199	0.075	2.675	0.007
**BO** → **GS**	0.103	0.103	0.072	1.434	0.152
**CM** → **GS**	0.261	0.261	0.066	3.964	0.000
**ES** → **GS**	0.026	0.027	0.071	0.369	0.712
**FI** → **GS**	0.090	0.090	0.074	1.223	0.221
**IT** → **GS**	0.135	0.137	0.079	1.713	0.087
**PR** → **GS**	0.111	0.110	0.071	1.567	0.117

Other relationships, such as BO → GS (0.103), ES → GS (0.026), FI → GS (0.090), IT → GS (0.135), and PR → GS (0.111), are also reported with their respective statistical values.

These results are provided for descriptive purposes to support further analysis by future researchers.

It should be noted that the purpose of this dataset is to provide structured data and measurement properties. Therefore, no causal interpretations or hypothesis testing conclusions are drawn in this manuscript. The reported statistics are presented solely to describe the dataset and its potential for reuse.

Table 6 evaluates the model fit based on several indicators, including SRMR, d_ULS, d_G, Chi-square, and NFI, for both the saturated and estimated models. The results show that the SRMR value of 0.046 for both models is below the threshold of 0.08, indicating a good model fit. Other indicators, such as d_ULS (2.295) and d_G (1.020), suggest an acceptable model fit distance, although these values do not have specific interpretation standards. The Chi-square value of 1446.252 represents the model fit; however, further interpretation requires additional information, such as degrees of freedom. The NFI value of 0.798 indicates that the model fit is in the moderate category. This value is below the commonly accepted standard of 0.90 for assessing a good model fit, suggesting room for improvement. Overall, the model demonstrates a fairly good level of fit, with the SRMR values meeting the criteria. However, some indicators, such as NFI, could be enhanced to improve the overall model fit.

**Table 6:** Model fit.

	Saturated model	Estimated model
**SRMR**	0.046	0.046
**d_ULS**	2.295	2.295
**d_G**	1.020	1.020
**Chi-square**	1446.252	1446.252
**NFI**	0.798	0.798

## 4 Experimental Design, Materials and Methods

The design of the questionnaire was meticulously developed through an extensive literature review, focusing specifically on theoretical and practical aspects of Green IT relevant to the higher education sector in Indonesia. This comprehensive background research ensured that each question was robust, relevant, and capable of eliciting meaningful insights about Green IT practices. To capture the breadth of perspectives and to assess the depth of understanding and challenges of implementing Green IT, the questionnaire exclusively employed Likert scales. This methodological choice facilitated the structured and standardized measurement of attitudes and perceptions across a broad spectrum of respondents. Likert scale questions were carefully constructed to evaluate various dimensions of Green IT, such as awareness, compliance, implementation challenges, and perceived benefits.

Each Likert item was designed to gauge the intensity of agreement or disagreement with statements pertaining to Green IT, ranging from strong agreement to strong disagreement. This format was chosen to provide quantitative data that could be easily analyzed for trends, patterns, and correlations. By using a uniform scale across all questions, the data gathered would allow for a consistent and comparative analysis of respondents' attitudes towards Green IT initiatives. The absence of open-ended questions was a deliberate choice to streamline data analysis and focus on quantifiable metrics that could be easily compared and analyzed statistically. This approach aimed to reduce the variability that open-ended responses might introduce, ensuring that the data remained focused on specific aspects of Green IT as identified in the preliminary literature review. This would facilitate a clearer understanding of the specific factors that influence the adoption and effectiveness of Green IT practices in the higher education sector, based on a unified scale of measurement.

A total of 275 valid responses were collected and used for analysis. This sample size is considered adequate for Partial Least Squares Structural Equation Modeling (PLS-SEM). First, following the widely used 10-times rule, the minimum sample size should be at least ten times the maximum number of structural paths directed at any construct in the model. In this study, the maximum number of arrows pointing to a single endogenous construct (Green IT Success) is seven. Therefore, the minimum required sample size is 70, and the current sample size of 275 substantially exceeds this threshold. Second, from a statistical power perspective, a sample size of 275 is sufficient to detect medium effect sizes with a statistical power of 0.80 at a significance level of 0.05, as commonly recommended in behavioral research. Third, previous PLS-SEM studies in similar contexts typically employ sample sizes ranging from 100 to 300, indicating that the current sample falls within an acceptable and robust range for model estimation and hypothesis testing.

### 4.1 Data collection process

The data collection process utilized a secure online platform, enabling the dissemination of the survey across a broad range of higher education institutions throughout Indonesia. This digital method was crucial for achieving a wide distribution, essential for capturing a diverse array of insights from various academic environments.

Utilizing an online survey platform not only expanded the reach of the data obtained but also enhanced the response rate significantly. Academics could participate at their convenience, which likely increased the number of completed responses. Furthermore, this approach streamlined the data collection process. Responses were automatically formatted into a database, which drastically reduced the need for manual data entry and mitigated the risks of human error associated with this task.

This method of data collection ensured that the responses were standardized, leading to a dataset that was uniform and immediately ready for analytical procedures. The use of a digital platform also facilitated quick updates to the questionnaire if needed, based on interim reviews of the data collection process, ensuring the quality and relevance of the data gathered. Such a setup was not only efficient but also scalable, accommodating a large volume of data securely and effectively.

### 4.2 Data collection strategy

For participant selection, a random sampling technique was utilized to choose academics from a pre-existing database of individuals who have shown interest or possess expertise in IT and sustainability. This method was strategically chosen to ensure that the sample represented a wide spectrum of perspectives within the academic community concerned with Green IT. By randomly selecting participants across various demographic and professional categories such as gender, age group, and education level Green IT aimed to capture diverse insights and experiences.

This sampling approach not only bolstered the representativeness of the data's findings but also strengthened the validity of the data by minimizing selection bias. The diversity in the dataset was crucial for examining how different groups within the academic community perceive and engage with Green IT practices, thus allowing for a more comprehensive analysis of factors affecting Green IT implementation in higher education institutions. The random sampling method facilitated an equitable chance of participation for all eligible academics in the database, ensuring that the sample reflected the broader academic environment in Indonesia.

### 4.3 Management Data

In this data, rigorous data management practices were upheld to align with strict data protection regulations. Personal identifiers were promptly removed from the dataset during the initial stages of the data cleaning process to safeguard participant anonymity, ensuring that individual respondents could not be identified in any published results. This was a critical step in maintaining the trust and confidentiality promised to participants. Furthermore, the data was stored in a secure, encrypted format to prevent unauthorized access. Access to this encrypted data was strictly limited to the research team, who required it for analysis purposes. Such precautions were essential to prevent data breaches and ensure that the data remained intact and unaltered.

This meticulous approach to data management not only protected the participants' information but also reinforced the integrity of the data. By ensuring that the data was handled securely and ethically, the research team could deliver robust and reliable findings. These insights are valuable for understanding the dynamics of Green IT adoption in higher education and can significantly contribute to academic discourse. Additionally, the results of this data provide a solid foundation for policymakers and institutional leaders to develop strategies aimed at enhancing the implementation and effectiveness of Green IT initiatives in the educational sector. This structured and secure handling of data underscores the credibility of the research and its potential impact on policy and practice.

## Limitations

A key limitation of this data is the constrained timeframe for data collection, which resulted in a minimal number of respondents participating in the survey. This restricted timeframe limited our ability to gather a more expansive and diverse dataset, potentially impacting the breadth and applicability of our findings. Future efforts should focus on extending the duration for response collection, thereby allowing for a larger and more varied group of participants. Increased respondent engagement would not only improve the representativeness of the sample but also enhance our understanding of the varied influences on Green IT implementation across different university settings in Indonesia. Such an expanded sample would enable a clearer depiction of the heterogeneity in responses, providing deeper insights into the effective strategies and challenges faced in the adoption of Green IT within the higher education sector.

## Ethics Statement

All participants involved in this data provided their written, informed consent to participate. Participation was voluntary and could be withdrawn at any time. Participants remained anonymous and their responses were dealt with in confidence.

## CRediT Author Statement

**Uky Yudatama:** Conceptualisation, Investigation, Data curation, Writing-Reviewing and Editing, Project administration. **Pristi Sukmasetya:** Methodology, Investigation, Formal analysis, Writing-Original draft. **Agus Setiawan:** Conceptualisation, Methodology, Writing-Reviewing and Editing. **Endah Ratna Arumi:** Data curation, Validation, Writing-Reviewing and Editing.

## Declaration of Competing Interest

The authors declare that they have no known competing financial interests or personal relationships which have or could be perceived to have, influenced the work reported in this article.

## Data Availability

Mendeley DataDataset Green IT Adoption in Sector Indonesian Higher Education (Original data).
